# Strategic Syntheses of Vine and Wine Resveratrol Derivatives to Explore Their Effects on Cell Functions and Dysfunctions

**DOI:** 10.3390/diseases6040110

**Published:** 2018-12-11

**Authors:** Norbert Latruffe, Dominique Vervandier-Fasseur

**Affiliations:** 1 Biochemistry of the Peroxisome, Inflammation and Lipid Metabolism, EA 7270, Université de Bourgogne Franche-Comté, 6, boulevard Gabriel, 21078 DIJON CEDEX, France; norbert.latruffe@u-bourgogne.fr; 2 Institut de Chimie Moléculaire de l’Université de Bourgogne, ICMUB-UMR CNRS 6302, Université de Bourgogne Franche-Comté, 9, avenue A. Savary, 21078 DIJON CEDEX, France

**Keywords:** resveratrol derivatives, synthesis strategies, substituents phenyl rings, biological targets, efficacy towards diseases

## Abstract

*Trans*-resveratrol, the most well-known polyphenolic stilbenoid, is found in grapes and accordingly in wine and it is considered to be beneficial for human health, especially towards the aging-linked cell alterations by providing numerous biological activities, such as anti-oxidant, antitumoral, antiviral, anti-inflammatory, neuroprotective, and platelet anti-aggregation properties. Although *trans*-resveratrol is a promising molecule, it cannot be considered as a drug, due to its weak bio-availability and fast metabolism. To overcome these weaknesses, several research teams have undertaken the synthesis of innovative *trans*-resveratrol derivatives, with the aim to increase its solubility in water and pharmacological activities towards cell targets. The aim of this review is to show the chronological evolution over the last 25 years of different strategies to develop more efficient *trans*-resveratrol derivatives towards organism physiology and, therefore, to enhance various pharmacological activities. While the literature on the development of new synthetic derivatives is impressive, this review will focus on selected strategies regarding the substitution of *trans*-resveratrol phenyl rings, first with hydroxy, methoxy, and halogen groups, and next with functionalized substituents. The effects on cell functions and dysfunctions of interesting resveratrol analogs will be addressed in this review.

## 1. Introduction

**Figure 1:**
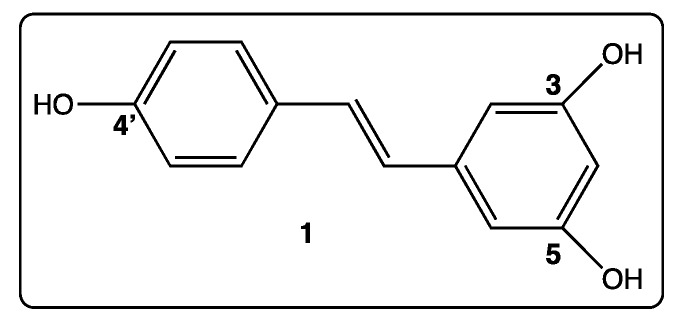
Structure of *trans*-resveratrol (**1**).

Polyphenolic compounds produced by vine belong essentially to flavonoids, stilbenoids, and anthocyanins, and are distributed in leaves, berries (seeds and skin), and lignified tissues. In the plant, they either play the role of phytoalexins (flavonoids and stilbenoids) [1,2] or are responsible for the color in leaves, flowers, and berries (anthocyanins) [3]. In addition, in each series, at least one polyphenolic compound provides health-promoting effects on humans. [4,5,6]. We were interested in *trans*-resveratrol (**1**, Figure 1), the leader in the polyphenolic stilbenoid series, present not only in vine, grapes, and, accordingly, in wine [7], but also in numerous other plants, including the Asiatic plant, *Polygonum cuspidatum* [8]; edible plants, such as peanuts [9]; and red fruit [10]. Accordingly, *trans*-resveratrol is part of our daily diet and this is a precious chance for our health because this molecule provides numerous biological activities, such as anti-oxidant [11], antitumoral [12], antiviral [13], and anti-inflammatory activities [14]. In addition, *trans*-resveratrol extents longevity [15], induces cell pro-differentiation [16,17], is a neuroprotective agent [18], and acts against platelet aggregation [19]. Cell targets have already been identified, such as membrane receptors, tyrosine kinases, phosphatases, sirtuins, and p53 anti-oncogene [20]. In addition, the interaction of resveratrol with tyrosyl transfer-RNA (tRNA) synthetase (TyrRS) may induce poly(ADP-ribose) polymerase 1 (PARP1) activation in cell nuclei in mice [21]. These various biological activities are often related to the anti-oxidant nature of resveratrol, itself explained in part by the ease of transfer of hydrogen atoms from the three phenolic groups to cellular species to act on adverse effects [22].

Since its discovery in 1940 [23], *trans*-resveratrol has been the subject of more than 20,000 publications that describe the different methods to obtain it (extraction from plants [24], synthetic ways [25], enzymatic syntheses [26]) and its numerous biological activities [27]. So, the regular consumption of food and moderated wine containing this health-beneficial molecule may be an effective way to prevent some diseases. In contrast, *trans*-resveratrol cannot be considered directly usable as a drug because of its weak bio-availability due to its low water solubility [28]. To overcome these difficulties, several research teams have undertaken the synthesis of new *trans*-resveratrol derivatives in the aim to enhance bio-availability and pharmacological activities. Previously, several reviews have stated a part of these studies by insisting either on synthetic schemes and biochemical activities [29] or on the pharmacological activities of new stilbene derivatives only [30,31,32,33]. Hence, this review will specifically focus on the chronological evolution for the last 25 years of different strategies followed by researchers to develop very efficient *trans*-resveratrol derivatives exhibiting various pharmacological activities. As in the case of *trans*-resveratrol, the numerous publications regarding synthetic *trans*-resveratrol derivatives are impressive. Indeed, a large panel of structural modifications could be achieved on the parent molecule, such as addressing the nature, the number, and the position of the phenyl rings’ substituents, the nature of the aryl ring, i.e., phenyl vs replacement of a phenyl ring by another aromatic one, the replacement of the C=C double bond by a diazo or imine bond, or an isosteric heterocyclic ring. It turns out that it is difficult to list all the derivatives and their diverse biochemical activities in a single review. Hence, this review will specifically focus on pharmacological improvements resulting from structural modifications performed at the phenyl ring substituents.

## 2. Which Strategies to Modify *trans*-Resveratrol

**Figure 2:**
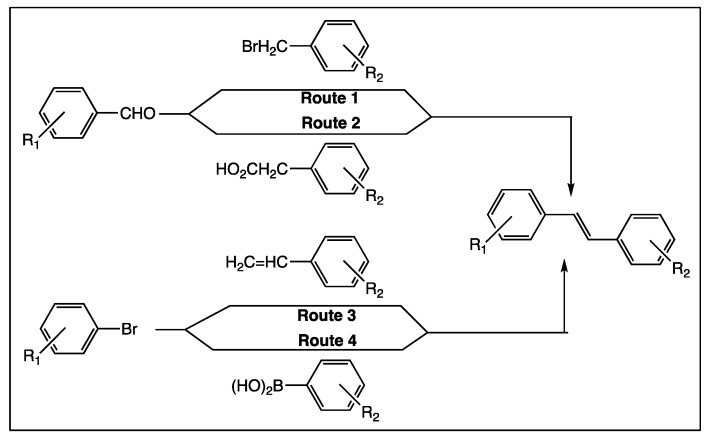
Principal synthetic methods for obtaining stilbene derivatives. Route 1: Wittig method [39,40], route 2: Perkin method [38], route 3: Heck method [39], route 4: Suzuki method [42].

The molecular structure of *trans*-resveratrol (**1**, Figure 1) is a stilbene core made of two phenyl rings linked by a double bond. Three hydroxy groups are present in both phenyl rings in position 3, 4′, and 5 (Figure 1). Their pKa values in aqueous medium are 9.8, 8.8, and 11.4, respectively [34]. The sensitive point of the molecule is the double bond separating the two phenyl rings that can be easily isomerized under light, knowing that isomer *E* of resveratrol is the biological active form [35]. Apart from this, *trans*-resveratrol is a non-toxic and air stable molecule, in the form of a white powder it has a melting at 261 °C; is soluble in ethanol, acetone, and tetrahydrofuran; and poorly soluble in water [36]. So, the chemical transformations of *trans*-resveratrol can be easily considered; they essentially take place at the phenolic functions that are transformed into ether or ester functions [37]. However, the access to new derivatives from *trans*-resveratrol itself sets limits to create innovative bio-active polyphenolic analogs. Fortunately, the essential stilbene core of resveratrol is easily accessible by different chemical methods, including Perkin [38], Wittig [39,40], Horner-Wittig-Emmons [41], Heck [39], and Suzuki [42] reactions (Figure 2). Each approach starts from different starting materials, which are usually commercially available and most of them are cheap. So, the library of *trans*-resveratrol derivatives that has been synthesized over the last 25 years is quite impressive. Over time, some new derivative structures have become more complex in order to move towards more selective and effective biological activities.

## 3. Phenyl Rings Substitution of *trans*-Resveratrol by Hydroxy, Methoxy, and Halogen Groups

**Figure 3:**
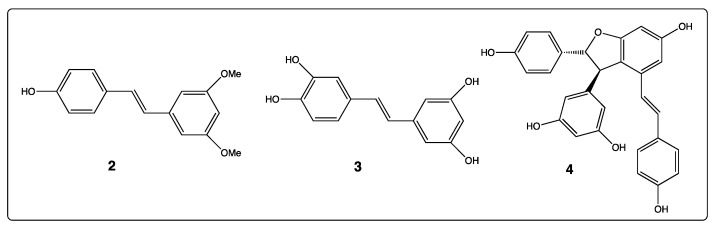
Structure of natural *trans*-resveratrol derivatives: Pterostilbene (**2**), piceatannol (**3**), and *trans*-ε-viniferin (**4**).

The biological activities of natural *trans*-resveratrol derivatives in vines, such as pterostilbene (**2**), piceatannol (**3**), and resveratrol oligomeric analogs as *trans*-ε-viniferin (**4**, Figure 3), are comparable to that of resveratrol (**1**) [43,44,45,46,47]. Thus, several research groups have used such bio-active molecules as an inspiration to synthesize numerous hydroxylated or/and methoxylated stilbenes [48,49,50].

Since the early 2000s, most research works have focused more specifically on non-natural resveratrol derivatives bearing hydroxy and/or methoxy groups and/or halogen atoms as substituents. Lately, a review summarized the manifold therapeutic activities of some of these polyphenolic derivatives [32]. In the conclusion, the authors of this review pointed out the fact that a structure-activity relationship study was missing. Indeed, it is difficult to predict pharmacological activities of this series of derivatives because changing one substituent may affect the biochemical property. In addition, as in the case of *trans*-resveratrol, one derivative may provide several biochemical properties. Thus, in this part, we will focus our discussion on a few examples of this type of resveratrol derivatives to illustrate the fact that it is often necessary to synthesize a large number of hydroxylated, methoxylated, and/or halogenated stilbenes to find good candidates for a particular therapy disease.

**Figure 4:**
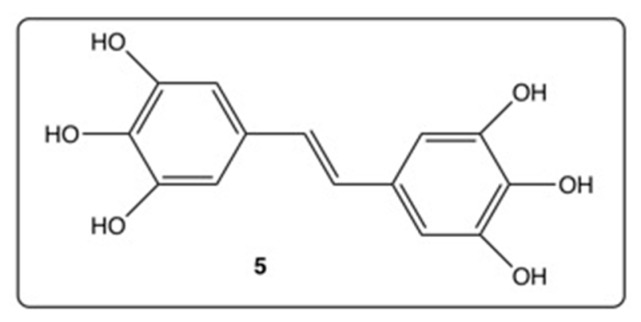
Structure of 3,4,5,3′,4′,5′-hexahydroxystilbene (**5**) bearing two pyrogallol groups.

Increasing the number of hydroxy groups on the resveratrol phenyl rings is already a good starting point to enhance pharmacological activities [48]. Thus, the two pyrogallol groups in 3,4,5,3′,4′,5′-hexahydroxystilbene (**5**, Figure 4) synthesized by Murias’s group appear to provide various activities for this resveratrol derivative, such as COX-2 inhibition correlated with a docking approach [51]; anti-oxidant activity through ortho semi-quinones formation [52], which triggers cytotoxic activity against breast cancer cells mediated by induction of p53 and downregulation of mitochondrial superoxide dismutase [53]; and oxidative stress in cancer cells [54]. Furthermore, resveratrol derivative **5** is a potent Human Immunodeficiency Virus (HIV-1) inhibitor at micromolar range [55].

**Figure 5:**
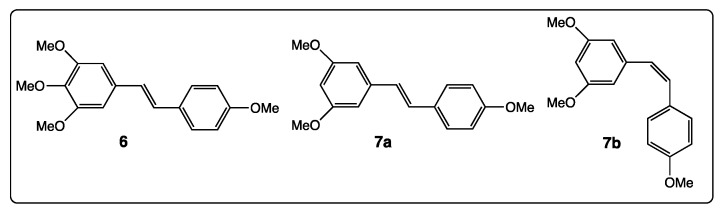
Structure of DMU-212 (**6**), *trans*-3,4′,5-trimethoxyresveratrol (**7a**), and cis-3,4′,5-trimethoxyresveratrol (**7b**).

In contrast, 3,4,5,4′-tetramethoxystilbene or DMU-212 (**6**, Figure 5) is only substituted by methoxy groups and may provide antitumoral activities, as described by different research groups. By selectively targeting the mitochondria of transformed lung fibroblasts, W138VA, DMU-212 (**6**) inhibited the cell growth (IC_50_ = 0.5 μM) compared with resveratrol (IC_50_ = 50 μM) [56]. Apoptotic induction and metastatic inhibition in melanoma cells by DMU-212 was highlighted too [57]. In in vivo experiments, injection of DMU-212 in male Wistar rats (rat hepatocarcinogenesis) allowed Murias’s group to prove that compound **6** may modulate the activation of NF-κB, AP-1, and STAT3 transcription factors [58]. Given the absence of hydroxy groups, an antioxidative activity cannot be invoked and the cell signaling pathway should be highlighted. By this way, it was found that another derivative bearing only methoxy groups, the *trans*-3,4′,5-trimethoxyresveratrol (**7a**, Figure 5), inhibited cancer cell growth (HeLa cells) by inhibiting tubulin polymerization [59]. In addition, the cis-3,4′,5-trimethoxyresveratrol (**7b**, Figure 5) was a very potent cell proliferation inhibitor and acted at the tubulin cholchicin binding site [60]. From these three last derivatives, **6**, **7a**, and **7b**, the presence of an additional methoxy group can modify the inhibition potencies, while the configuration of the double bond did not change it.

**Figure 6:**
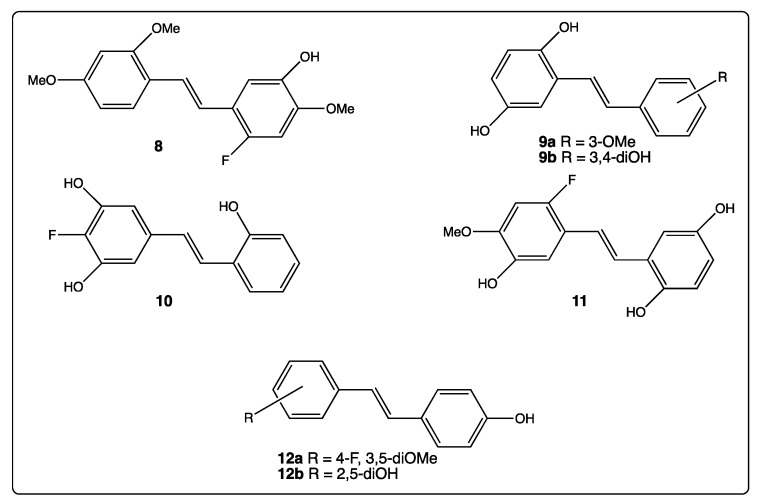
Structure of compounds **8**–**12**.

These two opposite examples of *trans*-resveratrol derivatives **5** and **6** show that hydroxy and methoxy groups may afford specific chemical properties, such as an improvement of the lipohilicity and bio-availability, promotion of interactions with amino acids in the receptor pocket [61,62], and induction of semi-quinones formation [52], which may induce specific pharmacological properties. Therefore, the combination of these two oxygenated groups, to which halogen atoms are possibly added, widens even more the field of pharmacological properties of these stilbenes. For example, Csuk’s team reported the biological activities of more than 100 stilbenes substituted with hydroxy and/or methoxy groups and/or fluorine atom only [63,64,65,66,67]. Throughout Csuk’s five publications, it appears that compounds **8**–**12** (Figure 6) provided antitumoral activity [64], acetylcholinesterase and butyrylcholinesterase inhibitions [65], anti-oxidant activity [66], and oxidant stress decrease in Caenorhabditis elegans [67].

**Figure 7:**
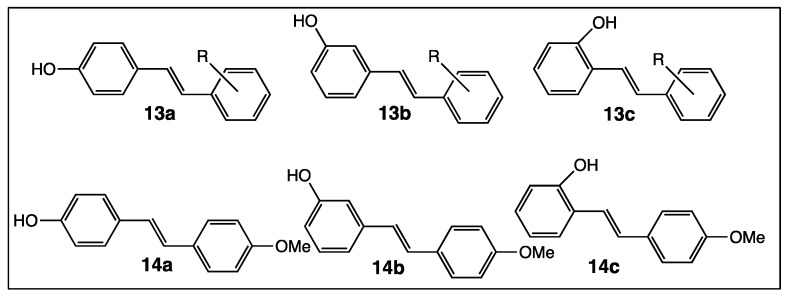
Structure of 4-hydroxy, 3-hydroxy, and 2-hydroxystilbenes **13a**–**c** and **14a**–**c**.

Generally, synthetic *trans*-resveratrol derivatives are tested for their potential therapeutic properties and rarely for their antimicrobial activities. However, among the library of stilbenoids of Csuk’s team, 25 compounds were evaluated for their antibacterial and antifungal activities [63]. They were divided in three series **13a**, **13b**, and **13c** in which the R substituent is a fluorine atom, or/and a hydroxy or a methoxy group (Figure 7). It turned out that position 4 with respect to the hydroxy group in compounds **13a** was more favorable than position 2 or 3 of the same group in compounds **13b** and **13c**.

In another study, 4-hydroxy-4′-methoxystilbene (**14a**, Figure 7) provided no antimicrobial activity towards two grapevine pathogens (Botrytis cinerea and Plasmopara viticola), while compounds **14b** and **14c** (Figure 7), both isomers of **14a**, showed an activity superior to those of *trans*-resveratrol and pterostilbene [68]. In contrast, in the case of antitumoral tests, the results are reversed: On the one hand, stilbene **14a** appeared to be a better candidate than *trans*-resveratrol for the inhibition of human colorectal tumor cells SW480, and on the other hand, isomer **14b** showed a weaker activity than the parent molecule [69].

## 4. Phenyl Rings Substitution of *trans*-Resveratrol by Functionalized Groups or Bioactive Moieties

The selected examples mentioned in the previous part show too many possibilities to dream up new *trans*-resveratrol derivatives as well as difficulties to predict their pharmacological activities. Since the late 2000s, several studies have shown that, while keeping the basic structure of *trans*-resveratrol, it remains possible to develop interesting derivatives starting from resveratrol (bioactivities of which are well defined) and adding judicious moieties, enabling enhancement of the bio-availability or to increase a particular biochemical property. As a result, therapeutic activities of these *trans*-resveratrol derivatives are better targeted.

**Figure 8:**
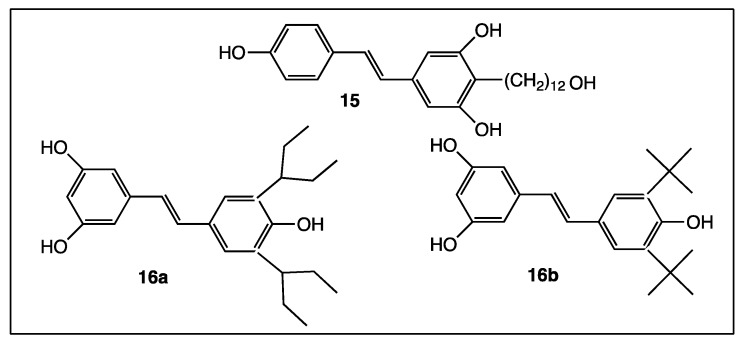
Structure of Resveratrol Fatty Alcohol (RFAs) (**15**) and compounds **16a** and **16b**.

Few examples of *trans*-resveratrol derivatives directly substituted on one of the aromatic carbon atoms have been described. Indeed, these substitution reactions cannot be carried out directly on *trans*-resveratrol itself and their syntheses require several chemical steps. However, a hybrid compound **15** named resveratrol fatty alcohol or RFAs (Figure 8) reported in 2007 results from the combination of a fatty alcohol and *trans*-resveratrol, which have neuroregenerative activity and neuroprotective features, respectively [70]. Cumulative effects at both parts in conjugate **15** provided a higher bio-activity than its parent moieties, polyphenol and fatty alcohol. In an inventive study [71], the *trans*-resveratrol structure was preserved and both ortho positions of 4-hydroxy group (responsible of anti-oxidant activity) were substituted with bulky electron donating groups in **16a** and **16b** (Figure 8). Adding two bulky substituents to the *trans*-resveratrol structure allowed enhancement of the anti-oxidant activity while strongly reducing interferences with estrogen and ArH receptors [71].

**Figure 9:**
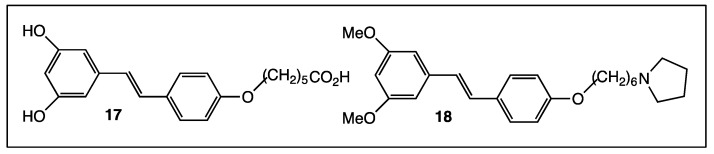
Structure of resveratrol aliphatic acid **17** and resveratrol aliphatic amine **18**.

The presence of chemical functions, such as ethers, carboxylic acids, esters, and amides, on the *trans*-resveratrol core may modify its lipophilic character and induce mechanisms in the cellular environment, which leads to the provision of better biological activities. The addition of various functions or alkyl chains could be carried out directly by *O*-acylation or *O*-alkylation reactions of commercially available *trans*-resveratrol. Therefore, the simple resveratrol aliphatic acid **17** (Figure 9) is more soluble in water than the parent molecule and inhibits the expression of TLR-2 [72]. Pterostilbene aliphatic amine **18** (Figure 9) was considered as a multitarget-directed agent for the therapy of the Alzheimer’s disease because it induced inhibition, although at a micromolar range of Aβ aggregation, and displayed moderate cholinesterase inhibition activity and acceptable inhibitory activity towards MonoAmine Oxidase (MAO) [73].

**Figure 10:**
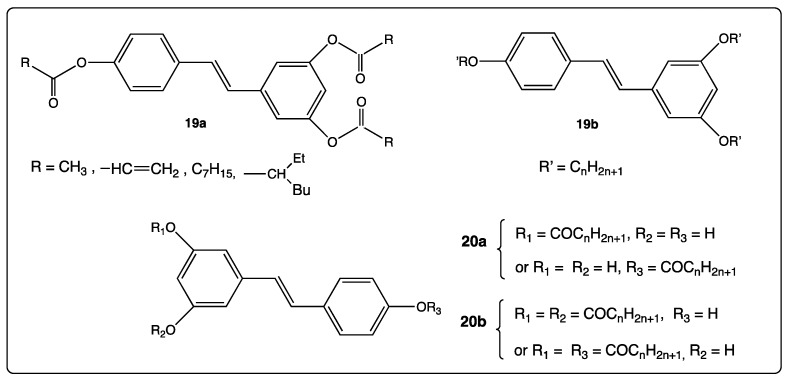
Structure of mono, di, tri ethers and esters **19a**–**b** and **20a**–**b**.

The bio-availability of *trans*-resveratrol was enhanced upon its transformation into tri-esters **19a** and tri-ethers **19b** (Figure 10) [74]. Improvement of this feature in these compounds led to a therapeutic interest (melanogenesis inhibition) and cosmetics application. Mono and diesters resveratrol derivatives **20a** and **20b** (Figure 10) were recently evaluated for their anti-oxidant activity and their possible use in food and biochemical systems [75]. While referencing to Biasutto’s work [76], the authors suggested that upon crossing the cell membrane barrier, the esters were hydrolyzed thus releasing resveratrol, which acts as an antioxidant agent.

**Figure 11:**
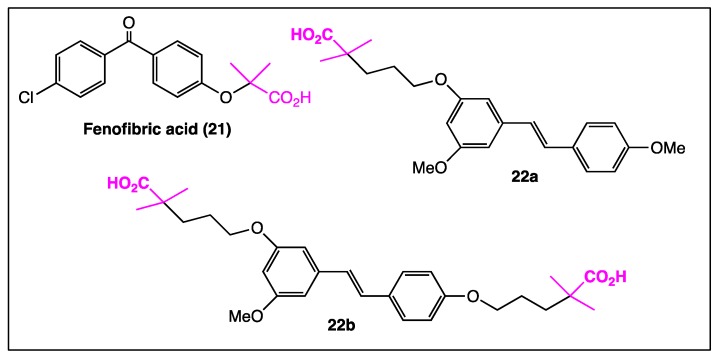
Structure of fenofibric acid (**21**) and hybrid compounds **22a**–**b**.

In the past ten years, the multi-targeted designed drugs (MTD’s) paradigm (that emerged especially in the fields of neurodegenerative diseases and cancers [77]) has consisted in designing hybrid compounds from at least two molecules providing complementary therapeutic activities. Hybridization of a rich bio-active molecule, such as *trans*-resveratrol, with a known pharmacophore has allowed researchers to better target biological activities. Given the good reactivity of phenolic functions, this concept has been easily applied to synthesize hybrid compounds. *O*-alkylation of one or two phenolic functions with a PPARα agonist, such as fenofibric acid (**21,** Figure 11), led to compounds **22a** and **22b** (Figure 11), lowering triglycerides in hyperlipidemic mice and blood glucose levels in KKAy mice, respectively [78].

**Figure 12:**
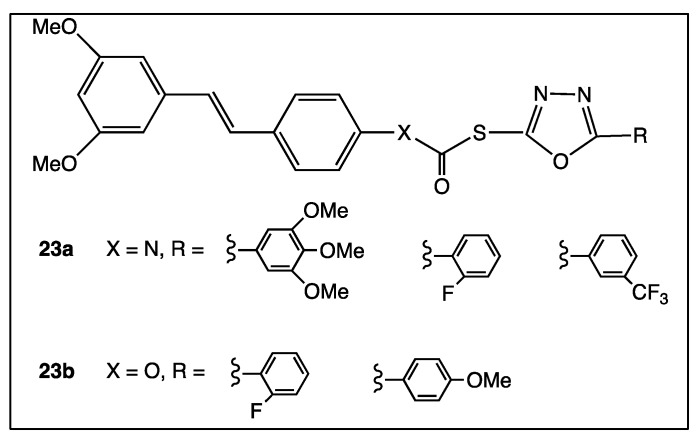
Structure of resveratrol-oxadiazole hybrid compounds **23a**–**b**.

1,3,4-Oxadiazole is a heterocyclic moiety with potential antitumoral activity if this one is part of a molecular bioactive structure. Therefore, hybridization of 1,3,4-oxadiazole and *trans*-resveratrol by an amide or an ester bond allowed Murty’s group to develop an inventive series of drug-like molecules, including **23a** and **23b** (Figure 12), that provided a dual therapeutic effect towards human cancer cell lines, SiHa, MDA-MB-231, and PANC-1, which turned out to be higher than that of polyphenol [79].

**Figure 13:**
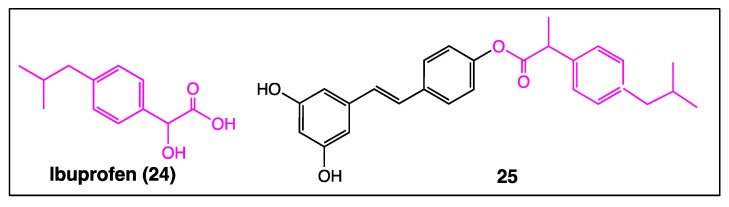
Structure of ibuprofen (**24**) and resveratrol-ibuprofen hybrid compound **25**.

The presence of a carboxylic group in nonsteroidal anti-inflammatory drugs, such as ibuprofen (**24,** Figure 13), is responsible for gastrointestinal toxicities. In contrast, *trans*-resveratrol has a protective effect against gastric mucosa damage. Therefore, linking this polyphenol and ibuprofen together by esterification reaction led to a hybrid compound **25** (Figure 13), which may solve gastrointestinal problems while keeping the anti-inflammatory activity of the ibuprofen moiety [80].

**Figure 14:**
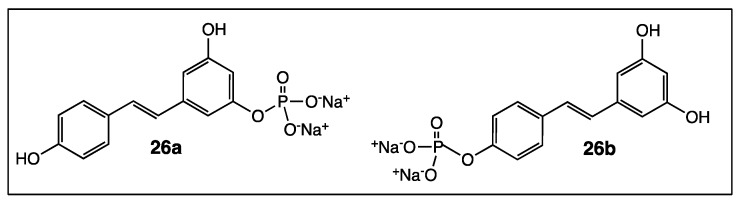
Structure of *O*-phosphorylresveratrol derivatives **26a**–**b**.

To mitigate the weak bio-availability and the unfavourable pharmacokinetic properties of *trans*-resveratrol, various bio-compatible resveratrol-loaded particles have been successfully developed [81]. Another way is to synthesize resveratrol derivatives bearing a moiety capable of promoting the crossing of the membrane barrier. In 2012, Sciuto’s team studied the interactions of two hydrophobic *O*-phosphorylresveratrol derivatives **26a** and **26b** (Figure 14) with a DMPC (1,2-Dimyristoyl-*sn*-glycero-3-phosphocholine) model membrane [82]. 3-*O*-phosphorylresveratrol derivative (**25a**) turned out to insert into the hydrophobic core of the membrane and diffused across it, while isomer **26b** was preferentially bound to the membrane surface and did not cross the membrane barrier. These results were correlated with the fact that the antitumoral effect of **26a** against DU-145 prostate cancer cells was higher than that of **26b**.

**Figure 15:**
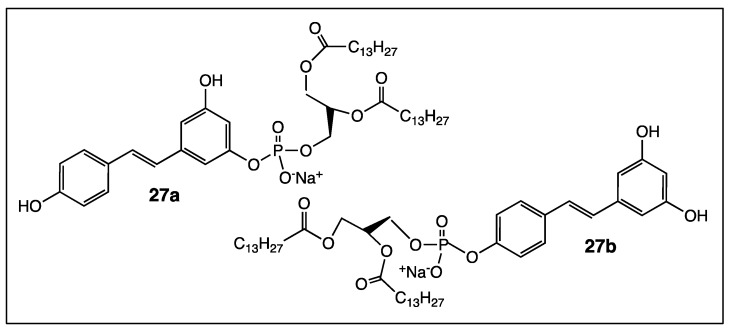
Structure of resveratrol-1,2-DiMyristoyl-*sn*-glycero-3-Phosphoholine (DMPC) hybrid compounds **27a**–**b**.

The same research team achieved the direct coupling of a lipophilic group (related to lipids membrane) to resveratrol derivatives **26a** and **26b** to afford amphiphilic resveratrol lipoconjugates **27a** and **27b** (Figure 15) [83]. These innovative *trans*-resveratrol derivatives had greater anticancer activity against the neuroblastoma SH-SY5Y cell line than the free parent molecule. Lately, a mixture of *O*-phosphorylresveratrol derivatives **26b** and amphiphilic resveratrol lipoconjugate **27b** was shown to be efficient to abolish hIAPP amyloid growth and membrane damage in diabetes mellitus type II pathology [84]. Both *trans*-resveratrol derivatives act in a complementary way to fight amyloid poration phenomena and lipid extraction by amyloid fibrils.

## 5. Discussion

The main goal of research teams in designing new resveratrol derivatives is the improvement of one or several biological activities of the parent molecule. These improvements involve not only the way to “dress” the stilbene scaffold, but also to address both the pharmacokinetics and bioavailability aspects. In this review, we only considered stilbene derivatives whose modifications relate to the nature, the number, and the position of aromatic rings’ substituents. We showed the chronological evolution over the last 25 years of different chemical strategies followed by researchers in the aim to develop efficient *trans*-resveratrol derivatives towards various pharmacological activities. It should be noted especially the evolution of derivatives bearing non-functionalized phenyl rings’ substituents to derivatives designed according to the multi-targeted designed drugs (MTD’s) paradigm. Because the number of publications related to such *trans*-resveratrol derivatives is impressive, the list of these relevant cited papers is far from exhaustive. However, among the selected examples in this review, the following conclusions may be raised based on the points summarized below.

First, even the number and position of the 9 hydroxy groups on resveratrol phenyl rings play an important role in the various activities of the polyphenols, and the presence of methoxy groups and/or halogen atoms may lead to interesting properties. Thus, the increase of the number of hydroxy groups on the resveratrol phenyl rings (Figure 4) enhances COX-2 inhibition, anti-oxidant activity, and cytotoxic effect against breast cancer [51,52,53,54]. Stilbenes **8**–**12** (Figure 6) bear both the hydroxy and methoxy groups and fluorine atoms that provide antitumoral activity [64], acetylcholinesterase and butyrylcholinesterase inhibition activities [60], and anti-oxidant activity [66]. In the other hand, in compound **14a**, the position 4 of the hydroxy group is less favorable than the positions 2 or 3 in compounds **14b** and **14c** (Figure 7) for antibacterial and antifungal activities [68] while the presence of the methoxy group and/or fluorine atom on the other phenyl ring of stilbene **13a** (Figure 7) reverses this result [69]. When 4-hydroxy group is surrounded by two bulky groups in stilbenes **16a** and **16b** (Figure 8), the anti-oxidant activity is enhanced, while strongly reducing its interferences with estrogen and ArH receptors [71]. However, the tetra-methoxylated stilbene DMU-212 (**6**, Figure 5) leads to an increase in antitumoral activity by apoptotic induction [56,57,58].

Thus, as a result of so many complex results, it appears that applying the multi-targeted designed drugs (MTD’s) paradigm may be a very promising concept to better identify judicious stilbene derivatives with interesting pharmacological activities. Indeed, innovative coupling of *trans*-resveratrol with a fatty alcohol provided resveratrol fatty alcohol or RFAs (**15**, Figure 8) bearing both neuroregenerative and neuroprotective features [70]. This hybrid compound may be considered as the premise of a large series of stilbenes designed according to the multi-targeted designed drugs (MTD’s) paradigm. Thus, the *O*-alkylation of one or two phenolic functions with a PPARα agonist, such as fenofibric acid (**21**), leads to hybrid compounds **22a** and **22b** (Figure 11), lowering triglycerides in hyperlipidemic mice and blood glucose levels in mice [78], respectively. Coupling ibuprofen (**24**) with resveratrol solves the side effect problem because the resveratrol moiety **25** (Figure 13) protects the gastric mucosa against the acidity of the anti-inflammatory drug [80].

In a last point, the lipophilic character of resveratrol is a crucial parameter to increase its biological activities. It can be modulated in one way or another depending on the nature of the added chemical functions (ethers, carboxylic acids, esters, amides, etc.) to the *trans*-resveratrol core. For example, a series of resveratrol aliphatic acids, including compound **17** (Figure 9), synthesized in 2008 proved to be more soluble in water than the parent molecule and, therefore, the binding affinity of **17** to human serum albumin was 40-fold higher [85]. It was recently shown that the mono-O-phosphorylresveratrol derivatives **26a** and **26b** (Figure 14) have a hydrophobic character. As a result, their interaction with DMPC model membrane turned out to be good [82]. In contrast, tri-esters **19a** and tri-ethers **19b** (Figure 10) have higher lipophilic characters than resveratrol and may be considered as good candidates for skin-whitening cosmetics [74].

Biochemical mechanisms and lipophilic aspects of resveratrol derivatives are overall well highlighted in the literature cited in this review. However, as it was mentioned in a recent review [33], we noticed that most of the biological tests carried out on resveratrol derivatives bearing hydroxy, methoxy, and halogen groups have been done on cultured cell lines (in vitro) or on isolated enzyme, but rarely in vivo and never through clinical studies. However, in vivo experiments with resveratrol derivatives bearing functionalized substituents have been carried out, but the pharmacokinetics aspects were not mentioned [78].

## 6. Conclusions

To get a better understanding of the biological effects of natural *trans*-resveratrol either from vine grape derived-beverages or from diet, the use of resveratrol derivatives appears very useful for the identification of cell targets to help maintain the best healthy conditions, e.g., to prevent diseases, such as stroke, cancer, and infection, and to increase longevity. Some resveratrol derivatives may allow differentiation of candidates with or without anti-oxidant properties. From a pharmaceutical point of view, the discovery of innovative resveratrol analogs is also very relevant to determine effective and safe dosage. Moreover, this requires more in vivo experiments to understand the metabolism of the derivatives and effects on whole organisms in terms of benefits and possible toxicity.
